# Whole exome sequencing identifies KCNH7 variants associated with epilepsy in children

**DOI:** 10.1016/j.gendis.2024.101322

**Published:** 2024-05-09

**Authors:** Fan Wu, Xinna Ji, Mengxiao Shen, Peidi Cheng, Shuo Feng, Yanyan Gao, Wanting Liu, Jinxiao Chen, Shupin Li, Xue Zhang, Qian Chen

**Affiliations:** a Department of Neurology, Children's Hospital Capital Institute of Pediatrics, Beijing 100020, China; b Chinese Academy of Medical Sciences & Peking Union Medical College, Beijing 100730, China; c State Key Laboratory of Medical Molecular Biology, Institute of Basic Medical Sciences, Chinese Academy of Medical Sciences & Peking Union Medical College, Beijing 100730, China

The KCNH7 (Potassium Voltage-Gated Channel Subfamily H Member 7) gene belongs to the Ether-A-Go-Go-Related Gene (ERG) subfamily of voltage-gated potassium channels. There are three members of the ERG family: ERG1, ERG2 and ERG3, with the latter being encoded by the KCNH7 gene. ERG1 is highly expressed in cardiomyocytes, and its mutations have been linked to arrhythmias, such as the long QT syndrome.^1^ Research on ERG2 is limited, while ERG3 is primarily expressed in neurons. The connection between the KCNH7 gene and neurological disorders remains to be elucidated.

We performed trio whole exome sequencing in a cohort of 975 pediatric epilepsy patients. Among probands with negative results, potential candidate genes were screened (see supplemental methods), and the KCNH7 gene was one of them, displaying *de novo* variants in three unrelated probands in the cohort. The detailed clinical manifestations are summarized in Table S1. Case 1 presented with both generalized tonic-clonic seizure and focal motor seizure; case 2 was diagnosed with West syndrome; and case 3 exhibited focal motor seizure with brain structural abnormality detected by magnetic resonance imaging (MRI). Case 2 and 3 experienced development regression. All three cases were seizure-free for at least one year, with the use of one to three antiseizure medications.

The three variants of the KCNH7 gene (NM_033272.4) were c.83A > G/p. K28R, c.1919A > G/p. E640G, and c.1324C > T/p. R442X. These variants had no recorded allele frequency in gnomAD. Statistical analysis revealed significant differences in aggregate frequencies of the mutant alleles between our cohort and controls in gnomAD (including all populations and East-Asian population) (3/1950 *vs*. 0/282730 in controls of gnomAD-all populations, *P* = 3.209 × 10^−7^; 3/1950 *vs*. 0/19946 in controls of gnomAD-East-Asian population, *P* = 0.001, respectively) (Table S2). The R442X in case 3 was a nonsense mutation, while the other two missense variants were predicted to be disease-causing by at least two of the following pathogenicity predicting tools recommended by the American College of Medical Genetics and Genomics: Mutation Taster, PolyPhen2, or Sorting Intolerant From Tolerant (SIFT). The amino acid sequence alignment indicated that K28R, E640G, and R442X were located on highly conserved residuals across various species (Fig. 1).

**Figure 1:**
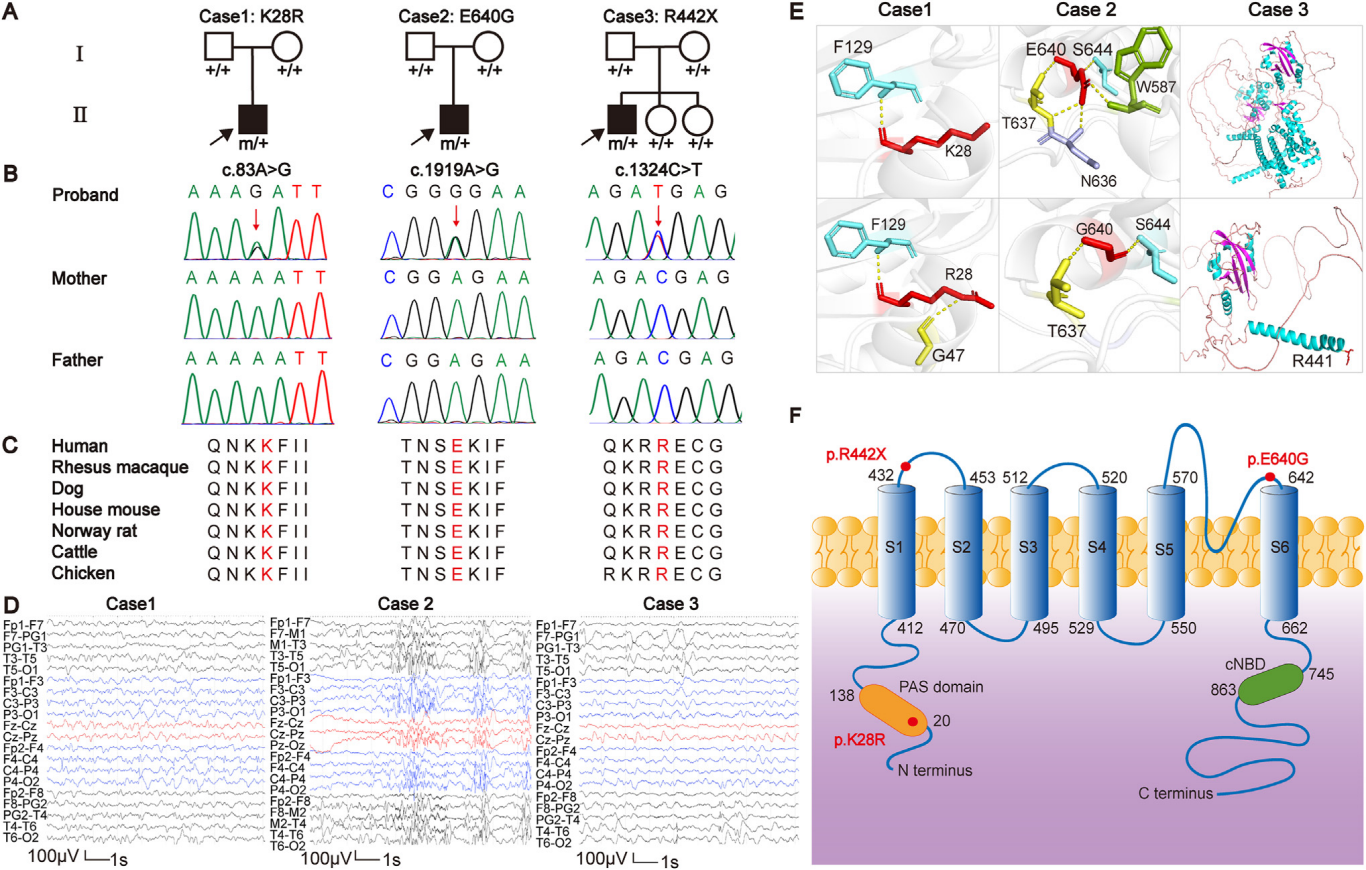
Genetic and clinical data of cases with KCNH7 variants. **(A)** Pedigrees of the cases with KCNH7 mutations. Patients with heterozygous mutation are indicated by m/+, while those negative for mutation are indicated by +/+. Probands are indicated by black arrows. **(B)** Sanger sequencing results of cases with KCNH7 mutations. The bases of the mutations are indicated with red arrows. **(C)** The amino acid sequences show that residues K28, E640, and R442 are highly conserved across various species. **(D)** Changes of interictal electroencephalography (EEG) in the cases with KCNH7 mutations. Interictal EEG in case 1 shows regional epileptiform discharges predominantly in the right central and middle temporal areas. Interictal EEG in case 2 shows hypsarrhythmia. Interictal EEG in case 3 shows bilateral temporal epileptiform discharges. **(E)** Schematic illustration of the changes in hydrogen bonds or three-dimensional construct of the protein. In case 1 and case 2, the residues where the mutations occurred are shown as red rods, and the hydrogen bonds are shown as yellow dotted lines. In case 3, the mutation leads to a protein truncation after R441, which is shown as a red rod. **(F)** Schematic diagram of ERG3 channel and the localization of the variants of KCNH7 identified in our cohort study. cNBD: cyclic nucleic binding homology domain; PAS: Per-Arnt-Sim.

To explore the potential influences of the three mutations on protein function, we initiated an analysis of the protein structure. Presently, the exact protein structure of ERG3 has not been described in the literature or protein databases. Therefore, we referred to the structure of ERG1, another member of the ERG family, which has been extensively studied, to predict the domains and spatial structure of the ERG3 protein using AlphaFold (https://alphafold.ebi.ac.uk/), SMART (http://smart.embl.de/) and DeepTMHMM (https://dtu.biolib.com/DeepTMHMM). The ERG3 protein has six transmembrane regions, S1to S6, with S1to S4 forming the voltage domain and S5 to S6 constituting the pore-forming domain. The region between S5 and S6 forms a pore for potassium ion passage in the tetrameric form of the ERG channel and plays a crucial role in cellular membrane potential repolarization.^2^ On the intracellular side, there are gating regulators: the Per-Arnt-Sim (PAS) domain and the cytosolic cyclic nucleic binding homology domain (cNBD). We then highlighted the mutation locations within these domains. As depicted in Figure 1, the K28R and E640G mutations led to alterations in the hydrogen bonds of the residues adjacent to the PAS domain and pore-forming domain, respectively, potentially affecting the steric configuration. The R442X mutation led to protein truncation between S1 and S2. It is noteworthy that these three mutations were located in positions seemingly exerting entirely distinct effects on the protein structure. This observation raised the question of whether the phenotypes of these cases also vary in severity based on different mutation locations.

Case 1, with the K28R mutation, had an onset of seizure at 15 months, and had three seizure events before receiving antiseizure medications. The brain MRI showed no abnormalities. Following the use of levetiracetam, the patient remained seizure-free for five years. He exhibited no developmental delay or intellectual disability. Case 2, carrying the E640G mutation, had the first seizure at the age of 5 months and was diagnosed with West syndrome. The brain MRI revealed no abnormalities. He was seizure-free for 18 months since the addition of vigabatrin. Initially, he showed developmental regression, and then exhibited catch-up development gradually. Case 3, with the R442X mutation, experienced the first seizure attack at 8 months with a frequency for up to five times a day. After administration of the third antiseizure medication, levetiracetam, he had been seizure-free for four years. The brain MRI revealed developmental abnormalities and brain atrophy. Developmental regression was also noted in this case. Collectively, the severity of the disease appeared to be consistent with the extent of the mutations’ impact on the protein structure. This association provided a foundation for further experimental and clinical investigations, contributing to the confirmation of the precise role of the KCNH7 gene in the etiology of epilepsy. It is worth noting that all three cases achieved seizure freedom, suggesting a potential association between KCNH7 variants and a favorable seizure prognosis. By analyzing BrainSpan transcriptomic data (www.brainspan.org), we observed that the expression level of the KCNH7 gene peaked around one year of age and remained relatively lower in subsequent years. This expression pattern may explain the early onset of seizures, and the favorable prognosis could be attributed to the decreased expression level of the KCNH7 gene.

Previous studies have suggested that the KCNH7 gene is potentially associated with various neurological and psychiatric disorders. Strauss et al’ s study^3^ identified a missense variant, c.1181G > A/p. R394H, in the KCNH7 gene as a potential candidate for bipolar spectrum disorder in 14 subjects. Another study, focusing on 262 probands with autism spectrum disorders, detected a *de novo* missense variant (c.2855T > G/p. I952R) in the KCNH7 gene in one patient.^4^ Interestingly, variants in the KCNH2 gene, which encodes ERG1, another member of the ERG family, have been reported in epilepsy patients, and predominantly located in the PAS domain and pore-forming domain.^1^ These findings were consistent with the affected domains observed in two of the KCNH7 variants reported in our study. Notably, the two candidate KCNH7 variants reported in previous researches were missense variants, whereas in our three cases, two were missense and one was protein-truncating mutation. As mentioned above, amino acid substitutions associated with missense mutations may lead to abnormalities in hydrogen bonding with adjacent residues, thereby impacting protein function. This indicates that alterations in protein function may serve as significant pathogenic factors, and the missense mutations in different domains may correlate with distinct phenotypes. Indeed, abnormalities in ion channel function are recognized as crucial pathogenic factors in epilepsy. Collectively, these findings suggested a potential role of the KCNH7 gene in epilepsy. However, until our study, there were limited clinical cases linking the KCNH7 gene to epilepsy.

Functional study of the KCNH7 gene provided evidence to support its association with epilepsy. In a study conducted by Xiao et al^5^ in 2018, the function of the ERG3 channel in mouse hippocampus was explored. The researchers observed an enhancement of neuronal intrinsic excitability and increased seizure susceptibility in *ERG3* knockdown mice. These findings provided additional support for the link between the KCNH7 gene and neuronal excitability, thus reinforcing its role in epileptogenesis. We are currently conducting relevant functional validation studies to further demonstrate the correlation between the KCNH7 gene and epilepsy.

In summary, our study provides evidence supporting that the KCNH7 gene is a candidate gene in pediatric epilepsy, and different domains affected by various mutations may be correlated with the severity of symptoms in patients. Further extensive clinical case studies and in-depth mechanistic research are essential to elucidate the pathogenic mechanisms of the KCNH7 gene variants.

## Ethics declaration

The ethical issues involved in this study were reviewed and approved by the Institutional Review Board of the Capital Institute of Pediatrics (No. SHERLL2022064) and informed consent was obtained from all patients’ parents.

## Author contributions

Conceptualization: Fan Wu, Xinna Ji, Mengxiao Shen, Peidi Cheng, Yanyan Gao, Wanting Liu, Xue Zhang, and Qian Chen; Methodology: Fan Wu, Xinna Ji, Mengxiao Shen, Peidi Cheng, and Jinxiao Chen; Software: Fan Wu, Xinna Ji, Mengxiao Shen, and Peidi Cheng; Validation: Fan Wu, Shuo Feng, and Xinna Ji; Formal Analysis: Fan Wu, and Jinxiao Chen; Investigation: Fan Wu, Mengxiao Shen, Peidi Cheng, Yanyan Gao, Wanting Liu, Jinxiao Chen, Shuo Feng, Shupin Li, and Qian Chen; Data Curation, Fan Wu, Yanyan Gao, Wanting Liu, Shuo Feng, and Shupin Li; Writing – Original Draft Preparation, Fan Wu; Writing – Review & Editing, Xinna Ji, and Qian Chen; Supervision: Xinna Ji, Xue Zhang, and Qian Chen; Project Administration: Qian Chen; Funding Acquisition: Xinna Ji, Xue Zhang, and Qian Chen. All authors have read and agreed the final manuscript.

## Funding

This work was funded by 10.13039/501100012166National Key Research and Development Program of China (No. 2022YFC2703903), Capital's Funds for Health Improvement and Research (China) (No. 2022-2Z-2104), and Rare disease Research Project Fund of E-town cooperation and development foundation (China) (No. YCXJ-JZ-2023-017).

## Conflict of interests

The authors declared that they have no conflict of interests.

## References

[1] Sanchez-Conde F.G., Jimenez-Vazquez E.N., Auerbach D.S., Jones D.K. (2022). The ERG1 K^+^ channel and its role in neuronal health and disease. Front Mol Neurosci.

[2] Atalar F., Acuner T.T., Cine N. (2010). Two four-marker haplotypes on 7q36.1 region indicate that the potassium channel gene *HERG1* (KCNH2, Kv11.1) is related to schizophrenia: a case control study. Behav Brain Funct.

[3] Strauss K.A., Markx S., Georgi B. (2014). A population-based study of KCNH_7_ p.Arg394His and bipolar spectrum disorder. Hum Mol Genet.

[4] Takata A., Miyake N., Tsurusaki Y. (2018). Integrative analyses of *de novo* mutations provide deeper biological insights into autism spectrum disorder. Cell Rep.

[5] Xiao K., Sun Z., Jin X. (2018). ERG3 potassium channel-mediated suppression of neuronal intrinsic excitability and prevention of seizure generation in mice. J Physiol..

