# Supplementary material for: Whole exome sequencing identifies KCNH7 variants associated with epilepsy in children
Source: Genes Dis. 2024 May 9;12(2):101322. doi: 10.1016/j.gendis.2024.101322 (PMC11615581; doi:10.1016/j.gendis.2024.101322)
Supplement: Multimedia component 1 [file mmc1.docx]

**Supplementary methods**

**1. Study population**

Participants were enrolled at Children’s Hospital Capital Institute of Pediatrics from July 2015 to October 2023. The inclusion criteria were as follows: (1) age under 18 at recruitment, (2) diagnosed by pediatric neurologists, meeting the diagnostic criteria for epilepsy in accordance with the International League Against Epilepsy criteria (2017), (3) suspected of potential genetic causes and underwent trio whole exome sequencing (WES). Probands with epilepsy likely attributed to head trauma, brain tumors, central nervous system infections, cerebrovascular diseases, and other underlying acquired brain injuries were excluded. All of the participants accepted genetic testing in our genetic diagnostic laboratory. In total, there were 975 probands in this cohort.

**2. DNA sequencing and variant interpretation**

Blood samples were collected from all probands and their biological parents. Genomic DNA was extracted from peripheral blood using the TIANamp Blood DNA Kit (Tiangen, Beijing, China). Beginning with 200ng high quality genomic DNA, libraries were constructed using the KAPA HyperPlus Kits (Kapa Biosystems) following the manufacture’s instruction. The post-PCR library was then employed for exome capture by xGen®Exome Research Panel (IDT, IA, USA). The final enriched libraries underwent sequencing with 150-bp paired-end runs on Illumina Hiseq 4000/NovaSeq 6000 (Illumina, CA, USA). Each sample had an average coverage exceeding 100×, with over 96% of targets covered at 20×. All sequencing data met the read-quality control requirement of more than 80% of Q30.

Pathogenicity classification was conducted in accordance with the American College of Medical Genetics and Genomics guidelines for the interpretation of variants and the variant classification categories include pathogenic (P), likely pathogenic (LP), variants of uncertain significance (VUS), likely benign and benign. Genetic testing results were classified as diagnostic, possibly-diagnostic, and negative. Diagnostic result was defined as the presence of (1) one P/LP variants of a single gene of autosomal dominant (AD) inheritance, or (2) P/LP variants of a single gene of X-linked inheritance, or (3) two P/LP variants of two alleles within a single gene of autosomal recessive (AR) inheritance, or (4) a P/LP copy number variation. Possibly-diagnostic result was defined as the presence of (1) only VUS in consistent with AD or XL disorders, or (2) two VUS of two alleles within a single gene of AR disorders, or (3) one P/LP variant and one VUS in two alleles respectively within a single gene of AR disorders, or (4) CNVs evaluated as VUS. Negative results were defined as the absence of P/LP variants or VUS, or only one variant in one of the two alleles of AR inheritance. Further, potential candidate genes were identified based on: (1) patients with negative result. (2) de novo, homozygous, compound heterozygous, or hemizygous variant with unaffected parents, or other variants with co-segregation. (3) minor allele frequency in normal populations <0.005. (4) non-synonymous variant. (5) genes expressed in brain tissue. The KCNH7 gene was one of the candidate genes with variants in three unrelated probands in this cohort. Other potential candidate genes were not the targets of this study. All of the potential candidate variants were validated by Sanger sequencing.

**3. Statistical analysis**

The comparison of two proportions was performed by the chi-squared test. IBM SPSS statistics software version 26.0 were used in data analysis. Statistical significance was attributed if *P* < 0.05.

**Supplementary tables**

**Table S1.** Clinical features of the cases with KCNH7 mutations

|  | Case1 | Case2 | Case3 |
| --- | --- | --- | --- |
| Variant (NM_033272.4) | c.83A>G/ p. K28R, de novo | c.1919A>G/ p. E640G, de novo | c.1324C>T/ p. R442X, de novo |
| Gender | Male | Male | Male |
| Current age | 8 years | 2 years | 5 years |
| Onset age of seizure | 1 years 3 months | 5 months | 8 months |
| Seizure type | GTCS, focal motor | Epileptic spasm | Focal motor |
| Seizure frequency | GTCS 2 times, focal motor once | In cluster, 20-30 times/day | 5 times/day |
| Fever-related | No | No | No |
| Epilepsy syndrome | No | West syndrome | No |
| Ictal EEG | NA | Spasm, with generalized medium to high amplitude slow wave, spike and wave | NA |
| Interictal EEG | Regional epileptiform discharges predominantly in right central and middle temporal areas | Hypsarrhythmia | Bilateral temporal epileptiform discharges |
| Brain MRI | Normal | Normal | Long T1 and T2 signal around bilateral ventricle, atrophy in the bilateral cerebral and cerebellar hemispheres |
| Antiseizure medications | LEV | ACTH, VGB | OXC, TPM, LEV |
| Seizure outcomes | Seizure-free for 5 years | Seizure-free for 1 year and 6 months | Seizure-free for 4 years |
| Developmental delay | No | Development regression | Development regression |

ACTH, adrenocorticotropic hormone; EEG, electroencephalography; GTCS, generalized tonic-clonic seizure; LEV, levetiracetam; MRI, magnetic resonance imaging; NA, not available; OXC, oxcarbazepine; TPM, topiramate; VGB, vigabatrin.

**Table S2** Gene-based burden analysis for KCNH7 mutations

|  | Allele count/number in this study | Allele count/number in gnomAD: all populations | Allele count/number in gnomAD: East-Asian population |
| --- | --- | --- | --- |
| **Identified KCNH7 mutations** |  |  |  |
| Chr2:163693271 (c.83A>G) | 1/1950 | -/- | -/- |
| Chr2:163291743 (c.1919A>G) | 1/1950 | -/- | -/- |
| Chr2:163302758 (c.1324C>T) | 1/1950 | -/- | -/- |
| **Total** | 3/1950 | 0/282730 | 0/19946 |
| **P value** |  | 3.209×10^-7^ | 0.001 |
